# Fibrinogen–albumin ratio predicting major adverse cardiovascular outcomes post‐percutaneous coronary intervention: A systematic review and exploratory meta‐analysis

**DOI:** 10.1002/clc.23981

**Published:** 2023-02-01

**Authors:** Rupak Desai, Kahtan Fadah, Sashwath Srikanth, N. F. N. Neha, Akhil Jain

**Affiliations:** ^1^ Independent Outcomes Researcher Atlanta Georgia USA; ^2^ Department of Internal Medicine Texas Tech University Health Sciences Center El Paso Texas USA; ^3^ Department of Internal Medicine East Carolina University ‐ Vidant Medical Center Greenville North Carolina USA; ^4^ Department of Medicine Uzhhorod National University Uzhhorod Ukraine; ^5^ Department of Internal Medicine Mercy Catholic Medical Center Darby Pennsylvania USA

To the Editor,

Progressive atherosclerosis involving inflammation and oxidative stress are the major pathophysiological process in the evolution of coronary artery disease (CAD).^1^, ^2^ Inflammatory markers have been associated with worse cardiovascular outcomes.^3^ High levels of fibrinogen, a key component of coagulation pathway and acute‐phase reactant, are thought to drive inflammation and atherosclerotic plaque process by stimulating the endothelial cell injury through the release of growth factors and smooth muscle growth causing stenosis or no‐flow.^3^, ^4^ Elevated serum fibrinogen level was independently associated with CAD severity.^4^ Furthermore, low albumin level has been reported to be associated with no‐reflow phenomenon.^5^ Hypoalbuminemia has been associated with higher risk of in‐hospital cardiogenic shock, resuscitated cardiac arrest and death.^6^ Albumin deficiency prevents platelet inhibition and leads to inflammatory and oxidative injuries.^2^ Fibrinogen–albumin ratio (FAR) has been lately used as a marker to predict the severity of CAD and is reported to be superior to fibrinogen or albumin alone.^7^ Several prospective and retrospective studies have proposed to use FAR as a prognostic factor for patients who underwent PCI.^1^, ^4^, ^5^, ^6^, ^7^, ^8^ The objective of this meta‐analysis is to assess whether FAR can be used to project in‐hospital short and thereafter long‐term outcomes of PCI following CAD or myocardial infarction (MI).

We systematically searched PubMed/Medline, Embase, and Scopus databases through January 2021 using the following keywords: “fibrinogen albumin ratio,” “percutaneous coronary intervention,” “PCI,” “coronary artery disease,” “myocardial infarction,” and “cardiovascular events.” All prospective or retrospective studies reporting the association of FAR and post‐PCI outcomes were included. Studies in non‐English language or sample size <20 were excluded. Primary outcomes were major cardiovascular adverse events (MACE), all‐cause mortality (ACM), cardiac mortality, revascularization, and no‐reflow. Critical appraisal of studies was performed using Joanna Briggs Institute (JBI) appraisal tool for meta‐analyses of observational cohort or cross‐sectional studies (Supporting Information: Table 1). Publication bias was assessed using visual inspection of funnel plots. OpenMeta[Analyst] software was used for random effects models and *I* ^2^ statistics to assess pooled odds ratio and heterogeneity.

Ten studies with 11 751 patients (mean/median age range 59–75 years, females 27.7%) were included with follow‐up duration ranging from 30 days to 5 years. Population with higher fibrinogen and low albumin level (high FAR ratio) had higher MACE (OR [odds ratio]: 1.11, 95% CI [confidence interval]: 1.01–1.22, *p*_heterogeneity_ = .032), cardiac mortality (OR: 1.63, 95% CI: 1.27–2.09, p_heterogeneity_ < .001), no‐reflow (OR: 3.64, 95% CI: 1.56–8.49, *p*_heterogeneity_ = .003), and revascularization need (OR: 1.25, 95% CI: 1.00–1.57, *p*_heterogeneity_ = .046). The association between FAR and ACM was not statistically significant (OR: 1.03, 95% CI: 0.997–1.064, *p*_heterogeneity_ .079; Figure 1).

**Figure 1:**
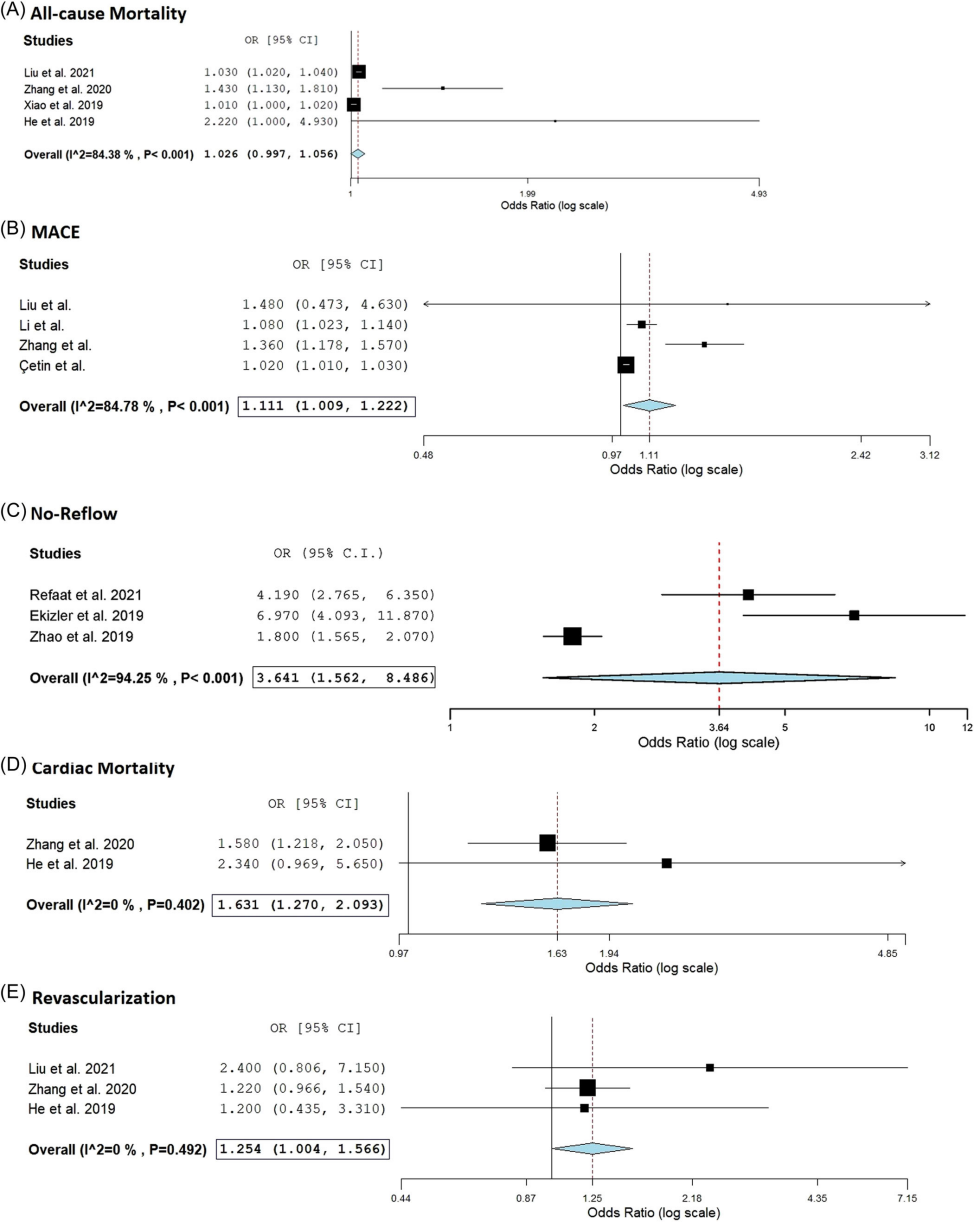
Fibrinogen–albumin ratio predicting post‐PCI cardiovascular outcomes: (A) all‐cause mortality, (B) MACE: major adverse cardiovascular outcomes, (C) no‐reflow phenomenon, (D) cardiac mortality, and (e) revascularization.

Our study is the first meta‐analysis to investigate the role of FAR in predicting short‐term in‐hospital and long‐term outcomes post‐PCI. In previous meta‐analyses, other biomarkers like red cell distribution width (RDW,) platelet‐to‐lymphocyte ratio (PLR), and neutrophil‐to‐lymphocyte ratio (NLR) were reported to be useful for predicting cardiovascular outcomes. Paucity of literature, known role of individual components in inflammation and its aftermaths, along with low cost, feasibility, and ease of obtaining FAR underpins the importance of evaluating the interplay of fibrinogen and albumin using pooled odds ratio in our meta‐analysis.

We observed significantly higher MACE, cardiac‐related fatality, and no‐reflow phenomenon with high FAR following PCI. Short‐term in‐hospital MACE may reflect upon the underlying intensity of cardiac compromise with CAD or MI, especially in the presence of a high comorbidity burden. Besides inflammatory setup in CAD/MI, traditional cardiovascular risk factors also have varying degrees of background inflammatory cascade that may or may not be modulated by guideline‐directed medical therapies. Though it is critical to acutely restore myocardial perfusion by recanalization of the culprit lesion in STEMI, 25% may experience a no‐reflow phenomenon without an obvious occluded vessel.^5^, ^8^ No‐reflow is linked to an increased risk of death, heart failure, and cardiac rupture as well as a larger infarction size, poorer left ventricular ejection fraction (LVEF), and unfavorable left ventricular remodeling.^5^ Cetin et al. revealed a strong correlation between MACE and higher FAR group in Acute coronary syndrome.^9^ Ekizler et al. performed a study of 617 STEMI patients, who underwent PCI in older adults with median age of 75 years old and found that increased level of FAR was higher in the no‐reflow group compared to the normal flow group.^10^ The no‐flow group was determined based on thrombolysis in myocardial infarction (TIMI) flow grades and TIMI < 3 in all these studies.^5^, ^8^, ^10^ Until now, revascularization has been examined as a secondary endpoint with a very small pool of patients. Overall, this meta‐analysis showed high FAR group may have a higher risk of requiring emergent‐unscheduled revascularization.^1^, ^2^, ^7^ ACM was mostly composed of cardiac‐related mortality. Global Registry of Acute Coronary Events (GRACE) score is an effective predictor of fatality (HR: 0.755, 95% CI: 0.723–0.790) in STEMI and adding FAR strengthened the statical significance (0.797, 95% CI: 0.741–0.819).^3^

Fibrinogen being the direct component of the coagulation cascade would correlate with the prothrombotic states and the underlying inflammatory milieu of thrombotic propagating pro‐inflammatory cytokines in acute MI or CAD. Albumin, synthesized in liver, has been established as an inversely proportional biomarker for inflammation with decreasing serum concentration in advancing inflammation. High FAR integrates two separate markers representing inflammation in its ratio, and therefore should be further investigated for its efficacy and sensitivity for cardiovascular outcomes on a short and long‐term basis and compared with previously explored biomarkers like RDW, platelet‐to‐lymphocyte, and neutrophil‐to‐lymphocyte.

A few limitations of this meta‐analysis included potential selection bias due to single‐center studies, lack of randomization allowing residual confounders, and lack of laboratory data on platelet activation, thrombosis, or coagulation parameters. Though all previous individual studies incorporating ACM demonstrated FAR as effective risk stratification, it was not statically significant in our meta‐analysis. We speculate sample size may have affected the pooled odds ratio. Racial differences in fibrinogen levels are reported earlier, however, in this meta‐analysis, FAR data in relation to demographics such as ethnicity and race were not described in included studies.^11^ To decrease such biases, the majority of the studies utilized a multivariable logistic regression model. Large‐randomized equitable prospective study might offer a more reliable assessment of FAR in all ethnic groups. At present, owing to the lack of such studies, our meta‐analysis provides critical clinical information on FAR's utility as a prognostic factor in determining cardiovascular events after PCI for CAD or MI.

In conclusion, FAR has become recently popular in assessing cardiovascular outcomes after PCI due to its accessibility, availability, and low cost. This meta‐analysis results suggest FAR may be utilized to predict MACE, cardiac mortality, risk of no flow, or need for revascularization in the post‐PCI population. Although ACM was not significantly associated with FAR in our study, we propose the need for prospective studies to guide clinical practice for the appropriate use of FAR as a risk‐stratification tool.

## CONFLICT OF INTEREST STATEMENT

The authors declare no conflict of interest.

## Supporting information

Supplementary information.
