# Supplementary material for: Fibrinogen–albumin ratio predicting major adverse cardiovascular outcomes post‐percutaneous coronary intervention: A systematic review and exploratory meta‐analysis
Source: Clin Cardiol. 2023 Feb 1;46(4):455–8. doi: 10.1002/clc.23981 (PMC10106656; doi:10.1002/clc.23981)
Supplement: Supplementary file 1 — Supplementary information. [file CLC-46-455-s001.docx]

| **Supplementary Table 1. Critical Appraisal of Included Studies** | | | | | | | | | | | | |
| --- | --- | --- | --- | --- | --- | --- | --- | --- | --- | --- | --- | --- |
| **First Author, Year** | **Type of Study Design** | **Sample representative of target population and selected from same population** | **Is sample size adequate (>20)?** | **Study subjects and settings described in detail** | **Exposure measures in valid and reliable way?** | **Were Confounding factors identified?** | **Were Strategies used (multivariable regression/matching) to deal with confounders?** | **Were groups free from outcomes at the start of study** | **Were outcomes measured in a valid and reliable way?** | **Was long-term follow-up time reported clearly?** | **Was follow-up complete for all patients or were there reasons to loss of follow-up? Were reasons mentioned clearly?** | **Were Appropriate statistical tests were used to report results?** |
| **Cetin, 2019** | Prospective Observational Cohort | Y | Y | Y | Y | Y | Y | Y | Y | NA | NA | Y |
| **Chen, 2020** | Retrospective Case Control | Y | Y | Y | Y | Y | Y | Y | Y | Y | Y | Y |
| **He, 2019** | Prospective Observational Cohort | Y | Y | Y | Y | Y | Y | Y | Y | NA | NA | Y |
| **Li, 2020** | Prospective Observational Cohort | Y | Y | Y | Y | Y | Y | Y | Y | Y | Y | Y |
| **Liu, 2021** | Prospective Observational Cohort | Y | Y | Y | Y | Y | Y | Y | Y | Y | Y | Y |
| **Refaat,2021** | Cross-sectional Observational Cohort | Y | Y | Y | Y | Y | Y | Y | Y | Y | Y | Y |
| **Xiao, 2019** | Retrospective Observational Cohort | Y | Y | Y | Y | Y | Y | Y | Y | Y | Y | Y |
| **Zhang, 2020** | Retrospective Observational Cohort | Y | Y | Y | Y | Y | Y | Y | Y | Y | Y | Y |
| **Zhao, 2019** | Retrospective Cross-sectional | Y | Y | Y | Y | Y | Y | Y | Y | NA | NA | Y |
| **Ekizler, 2019** | Retrospective Observational Cohort | NA | Y | Y | Y | Y | Y | Y | Y | NA | NA | Y |
